# Bis(3,5-di-*tert*-butyl-4*H*-1,2,4-triazol-4-amine-κ*N* ^1^)(nitrato-κ*O*)silver(I) ethanol monosolvate monohydrate

**DOI:** 10.1107/S1600536812019058

**Published:** 2012-05-05

**Authors:** Ya-Mei Liu, Jing-Huo Chen, Guang Yang, Seik Weng Ng

**Affiliations:** a Department of Chemistry, Zhengzhou University, Zhengzhou 450001, People’s Republic of China; b Department of Chemistry, University of Malaya, 50603 Kuala Lumpur, Malaysia; c Chemistry Department, Faculty of Science, King Abdulaziz University, PO Box 80203 Jeddah, Saudi Arabia

## Abstract

The Ag^I^ atom in the title compound, [Ag(NO_3_)(C_10_H_20_N_4_)_2_]·C_2_H_5_OH·H_2_O, is coordinated by the N atoms of two *N*-heterocycles [N—Ag—N = 151.5 (1)°]; the approximately linear coordination geometry is distorted into a T-shaped geometry owing to a long Ag⋯O_nitrate_ bond [2.717 (4) Å]. The N atoms of the *N*-heterocycles that are not involved in coordination point towards the lattice water mol­ecule, which functions as a hydrogen-bond donor. The water mol­ecule itself is a hydrogen-bond acceptor towards the ethanol solvent mol­ecule. Hydrogen bonds of the type N–H⋯O give rise to a layer motif parallel to (001).

## Related literature

For the synthesis of the *N*-heterocycle, see: Yang *et al.* (2012 ▶).
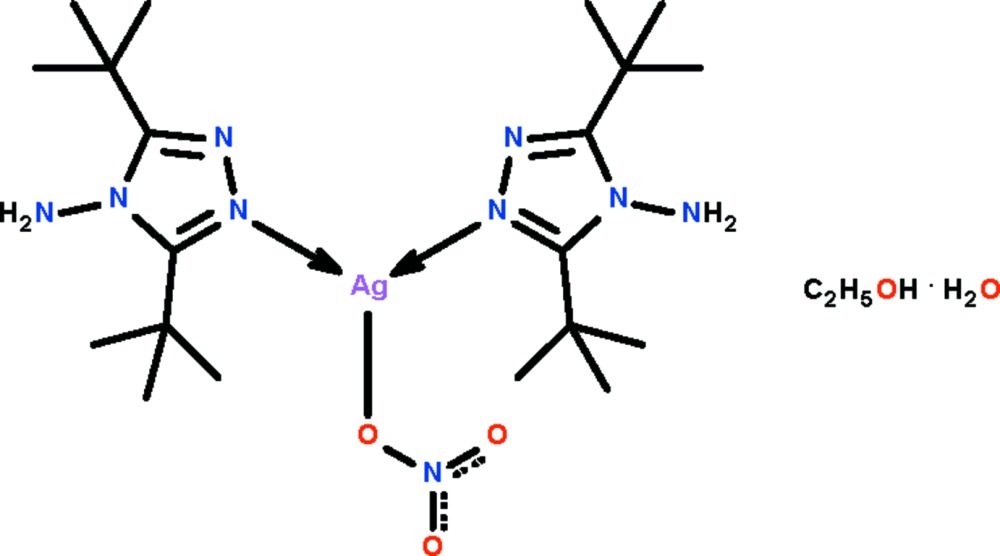


## Experimental

### 

#### Crystal data

[Ag(NO_3_)(C_10_H_20_N_4_)_2_]·C_2_H_6_O·H_2_O
*M* *_r_* = 626.56
Orthorhombic, 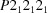

*a* = 10.149 (2) Å
*b* = 14.802 (3) Å
*c* = 20.405 (4) Å
*V* = 3065.3 (11) Å^3^
*Z* = 4
Mo *K*α radiation
μ = 0.70 mm^−1^
*T* = 293 K
0.25 × 0.20 × 0.15 mm

#### Data collection

Rigaku R-AXIS RAPID IP diffractometer
Absorption correction: multi-scan (*ABSCOR*; Higashi, 1995 ▶) *T*_min_ = 0.844, *T*_max_ = 0.902
11760 measured reflections
6724 independent reflections
5767 reflections with *I* > 2σ(*I*)
*R*_int_ = 0.044

#### Refinement

*R*[*F* ^2^ > 2σ(*F* ^2^)] = 0.054
*wR*(*F* ^2^) = 0.091
*S* = 1.15
6724 reflections
363 parameters
7 restraints
H atoms treated by a mixture of independent and constrained refinement
Δρ_max_ = 0.28 e Å^−3^
Δρ_min_ = −0.46 e Å^−3^
Absolute structure: Flack (1983 ▶), 2227 Friedel pairs
Flack parameter: 0.48 (3)

### 

Data collection: *RAPID-AUTO* (Rigaku, 1998 ▶); cell refinement: *RAPID-AUTO*; data reduction: *CrystalClear* (Rigaku/MSC, 2002 ▶); program(s) used to solve structure: *SHELXS97* (Sheldrick, 2008 ▶); program(s) used to refine structure: *SHELXL97* (Sheldrick, 2008 ▶); molecular graphics: *X-SEED* (Barbour, 2001 ▶); software used to prepare material for publication: *publCIF* (Westrip, 2010 ▶).

## Supplementary Material

Crystal structure: contains datablock(s) global, I. DOI: 10.1107/S1600536812019058/xu5525sup1.cif

Structure factors: contains datablock(s) I. DOI: 10.1107/S1600536812019058/xu5525Isup2.hkl

Additional supplementary materials:  crystallographic information; 3D view; checkCIF report

## Figures and Tables

**Table 1:** Hydrogen-bond geometry (Å, °)

*D*—H⋯*A*	*D*—H	H⋯*A*	*D*⋯*A*	*D*—H⋯*A*
O4—H4⋯O1w	0.85 (1)	1.94 (2)	2.772 (6)	167 (6)
O1w—H11⋯N2	0.84 (1)	2.16 (2)	2.976 (5)	164 (6)
O1w—H12⋯N6	0.84 (1)	2.08 (2)	2.915 (5)	171 (6)
N4—H41⋯O1^i^	0.88 (1)	2.20 (2)	3.008 (6)	153 (4)
N4—H42⋯O4^ii^	0.88 (1)	2.43 (2)	3.226 (6)	152 (4)
N8—H81⋯O2^iii^	0.88 (1)	2.27 (1)	3.144 (6)	171 (4)
N8—H82⋯O4^iv^	0.88 (1)	2.28 (2)	3.127 (7)	161 (6)

Symmetry codes: (i) 
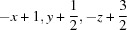
; (ii) 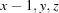; (iii) 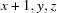; (iv) 
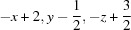
.

**Fig. 1.**
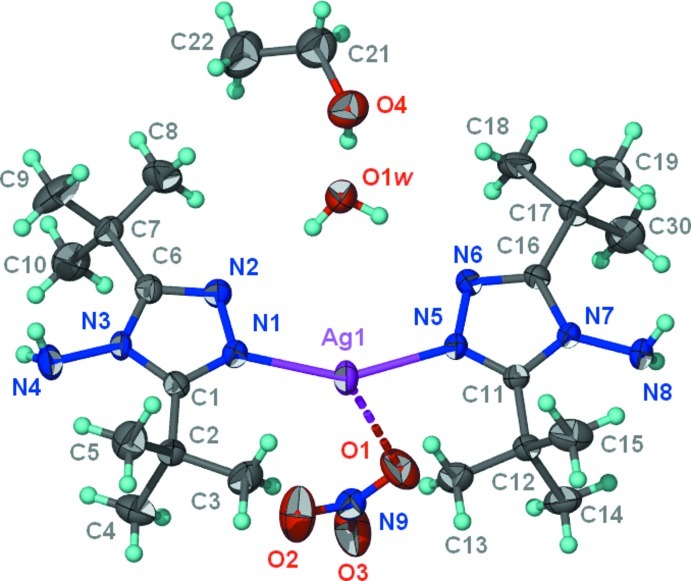
Thermal ellipsoid plot (Barbour, 2001) of Ag(NO3)(C10H20N4)2.H2O.C2H5OH at the 50% probability level; hydrogen atoms are drawn as spheres of arbitrary radius. The long Ag–O bond is denoted by a dashed bond.

## supplementary crystallographic information

### Comment

We recently reported the synthesis of di-*tert*-butyl-4*H*-1,2,4-triazol-4-amine, a compound that furnishes four isomeric silver(I) thiolatesadducts with silver(I) (Yang *et al.*, 2012). The Ag^I^ atom in Ag(NO_3_)(C_10_H_20_N_4_)_2_^.^H_2_O^.^C_2_H_5_OH (Scheme I) is coordinated by the the N atoms of two *N*-heterocycles [N–Ag–N 151.5 (1) °]; the approximately linear coordination geometry is distorted into a T-shaped geometry owing to a long Ag···O_nitrate_ bond [2.717 (4) Å] (Fig. 1). The N atoms of the *N*-heterocycles that are not involved in coordination point towards the water molecule, which functions as hydrogen-bond donor. The water molecule itself is hydrogen bond acceptor towards the ethanol molecule. Hydrogen bonds of the type N–H···O give rise to a layer motif (Table 1).

### Experimental

The *N*-heterocyclic amine was synthesized as reported (Yang *et al.*, 2012). An acetonitrile solution (1 ml) of silver nitrate (0.05 mmol, 8 mg) was mixed with an ethanol solution (1 ml) of the compound (0.01 mmol, 19 mg). The solution was set aside for the growth of colorless crystals, which were deposited after a week in 30% yield.

### Refinement

Carbon-bound H-atoms were placed in calculated positions (C–H 0.93 Å) and were included in the refinement in the riding model approximation, with *U*(H) set to 1.2*U*(C). The amino and water H-atoms were located in a difference Fourier map, and were refined with distance restraints N–H 0.88±0.01 Å, O–H 0.84±0.01 Å and H···H 1.37±0.01 Å; their temperature factors were refined.

### Crystal data

**Table:**

[Ag(NO_3_)(C_10_H_20_N_4_)_2_]·C_2_H_6_O·H_2_O	*F*(000) = 1320
*M**_r_* = 626.56	*D*_x_ = 1.358 Mg m^−^^3^
Orthorhombic, *P*2_1_2_1_2_1_	Mo *K*α radiation, λ = 0.71073 Å
Hall symbol: P 2ac 2ab	Cell parameters from 11783 reflections
*a* = 10.149 (2) Å	θ = 1.7–27.5°
*b* = 14.802 (3) Å	µ = 0.70 mm^−^^1^
*c* = 20.405 (4) Å	*T* = 293 K
*V* = 3065.3 (11) Å^3^	Prism, colorless
*Z* = 4	0.25 × 0.20 × 0.15 mm

### Data collection

**Table:**

Rigaku R-AXIS RAPID IP diffractometer	6724 independent reflections
Radiation source: fine-focus sealed tube	5767 reflections with *I* > 2σ(*I*)
Graphite monochromator	*R*_int_ = 0.044
ω scan	θ_max_ = 27.5°, θ_min_ = 1.7°
Absorption correction: multi-scan (*ABSCOR*; Higashi, 1995)	*h* = −13→13
*T*_min_ = 0.844, *T*_max_ = 0.902	*k* = 0→19
11760 measured reflections	*l* = −26→26

### Refinement

**Table:**

Refinement on *F*^2^	Secondary atom site location: difference Fourier map
Least-squares matrix: full	Hydrogen site location: inferred from neighbouring sites
*R*[*F*^2^ > 2σ(*F*^2^)] = 0.054	H atoms treated by a mixture of independent and constrained refinement
*wR*(*F*^2^) = 0.091	*w* = 1/[σ^2^(*F*_o_^2^) + (0.0195*P*)^2^ + 2.4088*P*] where *P* = (*F*_o_^2^ + 2*F*_c_^2^)/3
*S* = 1.15	(Δ/σ)_max_ = 0.001
6724 reflections	Δρ_max_ = 0.28 e Å^−^^3^
363 parameters	Δρ_min_ = −0.46 e Å^−^^3^
7 restraints	Absolute structure: Flack (1983), 2227 Friedel pairs
Primary atom site location: structure-invariant direct methods	Flack parameter: 0.48 (3)

### Fractional atomic coordinates and isotropic or equivalent isotropic displacement parameters (Å^2^)

**Table:**

	*x*	*y*	*z*	*U*_iso_*/*U*_eq_	
Ag1	0.66379 (3)	0.80143 (2)	0.790672 (16)	0.04839 (10)	
O1	0.6035 (3)	0.6499 (3)	0.8596 (2)	0.0729 (11)	
O2	0.4161 (4)	0.7157 (3)	0.8614 (2)	0.0849 (13)	
O3	0.4257 (4)	0.5733 (3)	0.8692 (3)	0.0876 (14)	
O4	0.9653 (4)	0.9402 (3)	0.59873 (19)	0.0669 (10)	
O1W	0.8036 (4)	0.7982 (4)	0.63591 (19)	0.0729 (11)	
N1	0.5190 (3)	0.8688 (2)	0.73091 (16)	0.0391 (8)	
N2	0.5237 (3)	0.8422 (2)	0.66564 (17)	0.0389 (8)	
N3	0.3520 (3)	0.9287 (2)	0.68103 (15)	0.0322 (7)	
N4	0.2395 (4)	0.9833 (3)	0.6723 (2)	0.0467 (10)	
N5	0.8624 (3)	0.7531 (2)	0.80776 (15)	0.0361 (8)	
N6	0.9183 (3)	0.7150 (3)	0.75210 (16)	0.0382 (9)	
N7	1.0358 (3)	0.6783 (2)	0.83740 (15)	0.0334 (8)	
N8	1.1297 (4)	0.6390 (3)	0.8800 (2)	0.0484 (11)	
N9	0.4821 (4)	0.6455 (3)	0.8638 (2)	0.0462 (9)	
C1	0.4162 (4)	0.9210 (3)	0.7396 (2)	0.0334 (9)	
C2	0.3745 (4)	0.9642 (3)	0.80379 (19)	0.0378 (10)	
C3	0.4758 (5)	0.9436 (4)	0.8568 (2)	0.0581 (14)	
H3A	0.5599	0.9676	0.8441	0.087*	
H3B	0.4828	0.8794	0.8625	0.087*	
H3C	0.4486	0.9708	0.8973	0.087*	
C4	0.2405 (5)	0.9256 (4)	0.8252 (3)	0.0625 (15)	
H4A	0.2471	0.8612	0.8297	0.094*	
H4B	0.1751	0.9400	0.7929	0.094*	
H4C	0.2156	0.9516	0.8665	0.094*	
C5	0.3651 (5)	1.0677 (3)	0.7967 (3)	0.0591 (13)	
H5A	0.4491	1.0913	0.7835	0.089*	
H5B	0.3402	1.0938	0.8379	0.089*	
H5C	0.3002	1.0825	0.7642	0.089*	
C6	0.4225 (4)	0.8794 (3)	0.6363 (2)	0.0362 (10)	
C7	0.3900 (4)	0.8649 (3)	0.5646 (2)	0.0403 (10)	
C8	0.5029 (5)	0.8112 (5)	0.5332 (2)	0.0709 (16)	
H8A	0.5136	0.7548	0.5559	0.106*	
H8B	0.5831	0.8454	0.5362	0.106*	
H8C	0.4829	0.7997	0.4880	0.106*	
C9	0.3738 (7)	0.9540 (4)	0.5276 (3)	0.077 (2)	
H9A	0.3036	0.9883	0.5470	0.115*	
H9B	0.3533	0.9418	0.4825	0.115*	
H9C	0.4543	0.9879	0.5301	0.115*	
C10	0.2645 (5)	0.8078 (4)	0.5584 (2)	0.0627 (14)	
H10A	0.2756	0.7522	0.5819	0.094*	
H10B	0.2481	0.7949	0.5130	0.094*	
H10C	0.1913	0.8406	0.5763	0.094*	
C11	0.9341 (4)	0.7314 (3)	0.8586 (2)	0.0321 (9)	
C12	0.9110 (4)	0.7612 (3)	0.9293 (2)	0.0375 (10)	
C13	0.7894 (5)	0.8214 (3)	0.9327 (2)	0.0550 (14)	
H13A	0.7137	0.7881	0.9181	0.083*	
H13B	0.8019	0.8732	0.9051	0.083*	
H13C	0.7761	0.8408	0.9771	0.083*	
C14	0.8880 (5)	0.6789 (3)	0.9731 (2)	0.0546 (13)	
H14A	0.8130	0.6458	0.9574	0.082*	
H14B	0.8721	0.6986	1.0172	0.082*	
H14C	0.9644	0.6407	0.9721	0.082*	
C15	1.0301 (5)	0.8155 (4)	0.9531 (2)	0.0621 (15)	
H15A	1.0158	0.8343	0.9976	0.093*	
H15B	1.0413	0.8678	0.9258	0.093*	
H15C	1.1077	0.7786	0.9508	0.093*	
C16	1.0224 (4)	0.6699 (3)	0.77094 (18)	0.0321 (9)	
C17	1.1144 (4)	0.6206 (3)	0.7243 (2)	0.0407 (11)	
C18	1.0508 (5)	0.6196 (4)	0.6563 (2)	0.0567 (14)	
H18A	1.0353	0.6805	0.6421	0.085*	
H18B	0.9686	0.5876	0.6583	0.085*	
H18C	1.1085	0.5900	0.6259	0.085*	
C19	1.2451 (4)	0.6716 (4)	0.7205 (3)	0.0661 (17)	
H19A	1.2855	0.6729	0.7630	0.099*	
H19B	1.2293	0.7323	0.7059	0.099*	
H19C	1.3027	0.6417	0.6901	0.099*	
C21	0.9561 (6)	0.9340 (5)	0.5300 (3)	0.0750 (17)	
H21A	0.9795	0.8732	0.5168	0.090*	
H21B	1.0200	0.9749	0.5107	0.090*	
C22	0.8266 (7)	0.9553 (5)	0.5036 (3)	0.093 (2)	
H22A	0.8286	0.9502	0.4567	0.139*	
H22B	0.8029	1.0158	0.5157	0.139*	
H22C	0.7629	0.9138	0.5210	0.139*	
C30	1.1378 (6)	0.5227 (4)	0.7462 (3)	0.0721 (17)	
H30A	1.1784	0.5223	0.7887	0.108*	
H30B	1.1945	0.4930	0.7153	0.108*	
H30C	1.0550	0.4914	0.7483	0.108*	
H4	0.918 (5)	0.900 (3)	0.616 (3)	0.07 (2)*	
H11	0.728 (3)	0.806 (4)	0.652 (3)	0.08 (2)*	
H12	0.844 (5)	0.775 (3)	0.6672 (19)	0.08 (2)*	
H41	0.267 (4)	1.032 (2)	0.652 (2)	0.053 (16)*	
H42	0.182 (3)	0.954 (2)	0.6480 (18)	0.047 (13)*	
H81	1.209 (2)	0.658 (3)	0.870 (2)	0.040 (14)*	
H82	1.118 (6)	0.5800 (10)	0.880 (3)	0.09 (2)*	

### Atomic displacement parameters (Å^2^)

**Table:**

	*U*^11^	*U*^22^	*U*^33^	*U*^12^	*U*^13^	*U*^23^
Ag1	0.04155 (16)	0.0608 (2)	0.04285 (16)	0.02041 (17)	−0.00957 (16)	−0.0024 (2)
O1	0.0377 (19)	0.094 (3)	0.087 (3)	−0.0074 (19)	0.0054 (19)	−0.010 (2)
O2	0.091 (3)	0.050 (2)	0.113 (4)	0.018 (2)	−0.032 (3)	−0.009 (2)
O3	0.053 (2)	0.059 (2)	0.151 (4)	−0.010 (2)	−0.013 (3)	0.028 (3)
O4	0.069 (3)	0.080 (3)	0.052 (2)	−0.010 (2)	−0.006 (2)	−0.005 (2)
O1W	0.050 (2)	0.110 (3)	0.059 (2)	0.016 (3)	0.0067 (18)	0.034 (3)
N1	0.0349 (18)	0.052 (2)	0.0309 (18)	0.0102 (17)	−0.0017 (14)	0.0019 (16)
N2	0.0382 (19)	0.047 (2)	0.0313 (18)	0.0093 (17)	−0.0015 (15)	−0.0042 (16)
N3	0.0285 (17)	0.0348 (17)	0.0332 (16)	0.0089 (16)	−0.0051 (15)	0.0004 (13)
N4	0.034 (2)	0.054 (3)	0.052 (3)	0.0135 (19)	−0.0080 (18)	−0.001 (2)
N5	0.0316 (18)	0.0479 (19)	0.0288 (17)	0.0124 (15)	−0.0002 (13)	0.0030 (14)
N6	0.0296 (16)	0.054 (2)	0.0314 (18)	0.0076 (17)	0.0007 (13)	0.0006 (17)
N7	0.0283 (16)	0.046 (2)	0.0255 (16)	0.0053 (15)	−0.0027 (13)	0.0033 (15)
N8	0.036 (2)	0.072 (3)	0.037 (2)	0.017 (2)	−0.0061 (16)	0.008 (2)
N9	0.048 (2)	0.053 (3)	0.038 (2)	0.004 (2)	−0.0045 (19)	−0.0014 (19)
C1	0.030 (2)	0.034 (2)	0.036 (2)	0.0032 (18)	0.0022 (17)	0.0019 (18)
C2	0.039 (2)	0.044 (2)	0.030 (2)	0.0069 (17)	0.0020 (17)	−0.0018 (18)
C3	0.068 (4)	0.072 (4)	0.034 (3)	0.010 (3)	−0.007 (3)	−0.008 (2)
C4	0.054 (3)	0.075 (4)	0.059 (3)	0.000 (3)	0.019 (3)	−0.001 (3)
C5	0.070 (3)	0.050 (3)	0.057 (3)	0.012 (2)	−0.006 (3)	−0.006 (3)
C6	0.033 (2)	0.035 (2)	0.041 (2)	−0.0013 (18)	0.0003 (18)	0.0038 (19)
C7	0.043 (2)	0.046 (3)	0.033 (2)	0.000 (2)	−0.0061 (18)	−0.001 (2)
C8	0.064 (3)	0.106 (5)	0.043 (3)	0.013 (4)	0.005 (2)	−0.018 (3)
C9	0.131 (6)	0.062 (3)	0.038 (3)	−0.001 (4)	−0.012 (3)	0.011 (2)
C10	0.061 (3)	0.075 (4)	0.053 (3)	−0.009 (3)	−0.010 (2)	−0.012 (3)
C11	0.0282 (19)	0.040 (2)	0.028 (2)	−0.0015 (17)	0.0000 (16)	0.0019 (17)
C12	0.035 (2)	0.048 (3)	0.029 (2)	0.002 (2)	0.0000 (17)	−0.0012 (19)
C13	0.072 (3)	0.058 (3)	0.035 (2)	0.022 (3)	0.007 (2)	−0.008 (2)
C14	0.067 (3)	0.056 (3)	0.041 (2)	0.009 (3)	0.015 (2)	0.009 (2)
C15	0.059 (3)	0.080 (4)	0.047 (3)	−0.023 (3)	0.005 (2)	−0.018 (3)
C16	0.0276 (19)	0.040 (2)	0.029 (2)	0.0008 (16)	0.0030 (15)	−0.0007 (16)
C17	0.034 (2)	0.056 (3)	0.032 (2)	0.0082 (19)	0.0064 (17)	−0.004 (2)
C18	0.053 (3)	0.086 (4)	0.031 (2)	0.001 (3)	0.010 (2)	−0.011 (2)
C19	0.037 (2)	0.111 (5)	0.051 (3)	−0.005 (3)	0.010 (2)	−0.009 (3)
C21	0.082 (4)	0.079 (4)	0.064 (4)	0.006 (3)	−0.003 (3)	−0.002 (3)
C22	0.086 (5)	0.111 (5)	0.081 (4)	−0.008 (5)	−0.023 (4)	0.012 (4)
C30	0.091 (5)	0.065 (3)	0.060 (3)	0.032 (3)	0.012 (3)	−0.005 (3)

### Geometric parameters (Å, º)

**Table:**

Ag1—N1	2.154 (3)	C7—C8	1.535 (7)
Ag1—N5	2.167 (3)	C8—H8A	0.9600
Ag1—O1	2.717 (4)	C8—H8B	0.9600
O1—N9	1.236 (5)	C8—H8C	0.9600
O2—N9	1.238 (5)	C9—H9A	0.9600
O3—N9	1.217 (5)	C9—H9B	0.9600
O4—C21	1.409 (7)	C9—H9C	0.9600
O4—H4	0.845 (10)	C10—H10A	0.9600
O1W—H11	0.836 (10)	C10—H10B	0.9600
O1W—H12	0.838 (10)	C10—H10C	0.9600
N1—C1	1.311 (5)	C11—C12	1.525 (6)
N1—N2	1.390 (5)	C12—C13	1.523 (6)
N2—C6	1.310 (5)	C12—C14	1.529 (6)
N3—C1	1.366 (5)	C12—C15	1.531 (6)
N3—C6	1.370 (5)	C13—H13A	0.9600
N3—N4	1.410 (5)	C13—H13B	0.9600
N4—H41	0.878 (10)	C13—H13C	0.9600
N4—H42	0.879 (10)	C14—H14A	0.9600
N5—C11	1.308 (5)	C14—H14B	0.9600
N5—N6	1.389 (4)	C14—H14C	0.9600
N6—C16	1.307 (5)	C15—H15A	0.9600
N7—C11	1.368 (5)	C15—H15B	0.9600
N7—C16	1.369 (5)	C15—H15C	0.9600
N7—N8	1.416 (5)	C16—C17	1.520 (5)
N8—H81	0.882 (10)	C17—C19	1.528 (6)
N8—H82	0.881 (10)	C17—C18	1.531 (6)
C1—C2	1.518 (6)	C17—C30	1.535 (7)
C2—C3	1.524 (6)	C18—H18A	0.9600
C2—C4	1.538 (6)	C18—H18B	0.9600
C2—C5	1.542 (6)	C18—H18C	0.9600
C3—H3A	0.9600	C19—H19A	0.9600
C3—H3B	0.9600	C19—H19B	0.9600
C3—H3C	0.9600	C19—H19C	0.9600
C4—H4A	0.9600	C21—C22	1.454 (8)
C4—H4B	0.9600	C21—H21A	0.9700
C4—H4C	0.9600	C21—H21B	0.9700
C5—H5A	0.9600	C22—H22A	0.9600
C5—H5B	0.9600	C22—H22B	0.9600
C5—H5C	0.9600	C22—H22C	0.9600
C6—C7	1.516 (6)	C30—H30A	0.9600
C7—C9	1.529 (7)	C30—H30B	0.9600
C7—C10	1.533 (6)	C30—H30C	0.9600
			
N1—Ag1—N5	151.45 (12)	H9A—C9—H9C	109.5
N1—Ag1—O1	121.43 (13)	H9B—C9—H9C	109.5
N5—Ag1—O1	81.58 (12)	C7—C10—H10A	109.5
N9—O1—Ag1	107.7 (3)	C7—C10—H10B	109.5
C21—O4—H4	109 (4)	H10A—C10—H10B	109.5
H11—O1W—H12	102 (6)	C7—C10—H10C	109.5
C1—N1—N2	108.9 (3)	H10A—C10—H10C	109.5
C1—N1—Ag1	137.7 (3)	H10B—C10—H10C	109.5
N2—N1—Ag1	112.8 (2)	N5—C11—N7	108.0 (3)
C6—N2—N1	107.0 (3)	N5—C11—C12	126.4 (4)
C1—N3—C6	106.8 (3)	N7—C11—C12	125.5 (3)
C1—N3—N4	123.0 (3)	C13—C12—C11	109.7 (3)
C6—N3—N4	130.1 (3)	C13—C12—C14	108.3 (4)
N3—N4—H41	106 (3)	C11—C12—C14	110.3 (4)
N3—N4—H42	109 (3)	C13—C12—C15	108.5 (4)
H41—N4—H42	110 (4)	C11—C12—C15	109.3 (3)
C11—N5—N6	108.8 (3)	C14—C12—C15	110.7 (4)
C11—N5—Ag1	136.6 (3)	C12—C13—H13A	109.5
N6—N5—Ag1	112.5 (2)	C12—C13—H13B	109.5
C16—N6—N5	107.3 (3)	H13A—C13—H13B	109.5
C11—N7—C16	106.9 (3)	C12—C13—H13C	109.5
C11—N7—N8	123.3 (3)	H13A—C13—H13C	109.5
C16—N7—N8	129.7 (4)	H13B—C13—H13C	109.5
N7—N8—H81	110 (3)	C12—C14—H14A	109.5
N7—N8—H82	108 (4)	C12—C14—H14B	109.5
H81—N8—H82	116 (5)	H14A—C14—H14B	109.5
O3—N9—O1	121.4 (4)	C12—C14—H14C	109.5
O3—N9—O2	119.1 (4)	H14A—C14—H14C	109.5
O1—N9—O2	119.5 (5)	H14B—C14—H14C	109.5
N1—C1—N3	108.1 (3)	C12—C15—H15A	109.5
N1—C1—C2	125.9 (4)	C12—C15—H15B	109.5
N3—C1—C2	125.9 (3)	H15A—C15—H15B	109.5
C1—C2—C3	109.9 (3)	C12—C15—H15C	109.5
C1—C2—C4	109.6 (4)	H15A—C15—H15C	109.5
C3—C2—C4	108.7 (4)	H15B—C15—H15C	109.5
C1—C2—C5	110.7 (4)	N6—C16—N7	109.0 (3)
C3—C2—C5	107.9 (4)	N6—C16—C17	123.9 (3)
C4—C2—C5	109.9 (4)	N7—C16—C17	127.0 (4)
C2—C3—H3A	109.5	C16—C17—C19	109.2 (4)
C2—C3—H3B	109.5	C16—C17—C18	108.2 (3)
H3A—C3—H3B	109.5	C19—C17—C18	108.9 (4)
C2—C3—H3C	109.5	C16—C17—C30	111.5 (4)
H3A—C3—H3C	109.5	C19—C17—C30	110.3 (4)
H3B—C3—H3C	109.5	C18—C17—C30	108.6 (4)
C2—C4—H4A	109.5	C17—C18—H18A	109.5
C2—C4—H4B	109.5	C17—C18—H18B	109.5
H4A—C4—H4B	109.5	H18A—C18—H18B	109.5
C2—C4—H4C	109.5	C17—C18—H18C	109.5
H4A—C4—H4C	109.5	H18A—C18—H18C	109.5
H4B—C4—H4C	109.5	H18B—C18—H18C	109.5
C2—C5—H5A	109.5	C17—C19—H19A	109.5
C2—C5—H5B	109.5	C17—C19—H19B	109.5
H5A—C5—H5B	109.5	H19A—C19—H19B	109.5
C2—C5—H5C	109.5	C17—C19—H19C	109.5
H5A—C5—H5C	109.5	H19A—C19—H19C	109.5
H5B—C5—H5C	109.5	H19B—C19—H19C	109.5
N2—C6—N3	109.2 (4)	O4—C21—C22	114.5 (6)
N2—C6—C7	123.5 (4)	O4—C21—H21A	108.6
N3—C6—C7	127.2 (4)	C22—C21—H21A	108.6
C6—C7—C9	112.1 (4)	O4—C21—H21B	108.6
C6—C7—C10	109.8 (4)	C22—C21—H21B	108.6
C9—C7—C10	110.2 (4)	H21A—C21—H21B	107.6
C6—C7—C8	108.2 (4)	C21—C22—H22A	109.5
C9—C7—C8	108.8 (4)	C21—C22—H22B	109.5
C10—C7—C8	107.5 (4)	H22A—C22—H22B	109.5
C7—C8—H8A	109.5	C21—C22—H22C	109.5
C7—C8—H8B	109.5	H22A—C22—H22C	109.5
H8A—C8—H8B	109.5	H22B—C22—H22C	109.5
C7—C8—H8C	109.5	C17—C30—H30A	109.5
H8A—C8—H8C	109.5	C17—C30—H30B	109.5
H8B—C8—H8C	109.5	H30A—C30—H30B	109.5
C7—C9—H9A	109.5	C17—C30—H30C	109.5
C7—C9—H9B	109.5	H30A—C30—H30C	109.5
H9A—C9—H9B	109.5	H30B—C30—H30C	109.5
C7—C9—H9C	109.5		
			
N1—Ag1—O1—N9	−17.7 (4)	C1—N3—C6—C7	178.5 (4)
N5—Ag1—O1—N9	−179.6 (3)	N4—N3—C6—C7	−5.4 (7)
N5—Ag1—N1—C1	−145.1 (4)	N2—C6—C7—C9	−126.9 (5)
O1—Ag1—N1—C1	75.1 (5)	N3—C6—C7—C9	55.8 (6)
N5—Ag1—N1—N2	44.9 (5)	N2—C6—C7—C10	110.1 (5)
O1—Ag1—N1—N2	−94.9 (3)	N3—C6—C7—C10	−67.1 (6)
C1—N1—N2—C6	−0.1 (5)	N2—C6—C7—C8	−7.0 (6)
Ag1—N1—N2—C6	172.9 (3)	N3—C6—C7—C8	175.8 (4)
N1—Ag1—N5—C11	157.9 (4)	N6—N5—C11—N7	−0.9 (4)
O1—Ag1—N5—C11	−55.9 (4)	Ag1—N5—C11—N7	160.4 (3)
N1—Ag1—N5—N6	−41.2 (4)	N6—N5—C11—C12	178.1 (4)
O1—Ag1—N5—N6	105.0 (3)	Ag1—N5—C11—C12	−20.6 (7)
C11—N5—N6—C16	0.8 (4)	C16—N7—C11—N5	0.7 (4)
Ag1—N5—N6—C16	−165.4 (3)	N8—N7—C11—N5	−177.0 (4)
Ag1—O1—N9—O3	152.1 (4)	C16—N7—C11—C12	−178.3 (4)
Ag1—O1—N9—O2	−26.7 (5)	N8—N7—C11—C12	4.0 (6)
N2—N1—C1—N3	0.6 (5)	N5—C11—C12—C13	0.0 (6)
Ag1—N1—C1—N3	−169.7 (3)	N7—C11—C12—C13	178.8 (4)
N2—N1—C1—C2	179.6 (4)	N5—C11—C12—C14	119.2 (5)
Ag1—N1—C1—C2	9.3 (7)	N7—C11—C12—C14	−61.9 (5)
C6—N3—C1—N1	−0.9 (5)	N5—C11—C12—C15	−118.8 (5)
N4—N3—C1—N1	−177.4 (4)	N7—C11—C12—C15	60.0 (5)
C6—N3—C1—C2	−179.9 (4)	N5—N6—C16—N7	−0.3 (5)
N4—N3—C1—C2	3.6 (6)	N5—N6—C16—C17	−177.8 (4)
N1—C1—C2—C3	4.1 (6)	C11—N7—C16—N6	−0.2 (5)
N3—C1—C2—C3	−177.1 (4)	N8—N7—C16—N6	177.3 (4)
N1—C1—C2—C4	−115.3 (5)	C11—N7—C16—C17	177.2 (4)
N3—C1—C2—C4	63.5 (6)	N8—N7—C16—C17	−5.3 (7)
N1—C1—C2—C5	123.2 (5)	N6—C16—C17—C19	108.7 (5)
N3—C1—C2—C5	−58.0 (6)	N7—C16—C17—C19	−68.4 (6)
N1—N2—C6—N3	−0.5 (5)	N6—C16—C17—C18	−9.8 (6)
N1—N2—C6—C7	−178.2 (4)	N7—C16—C17—C18	173.1 (4)
C1—N3—C6—N2	0.9 (5)	N6—C16—C17—C30	−129.2 (5)
N4—N3—C6—N2	177.0 (4)	N7—C16—C17—C30	53.8 (6)

### Hydrogen-bond geometry (Å, º)

**Table:**

*D*—H···*A*	*D*—H	H···*A*	*D*···*A*	*D*—H···*A*
O4—H4···O1w	0.85 (1)	1.94 (2)	2.772 (6)	167 (6)
O1w—H11···N2	0.84 (1)	2.16 (2)	2.976 (5)	164 (6)
O1w—H12···N6	0.84 (1)	2.08 (2)	2.915 (5)	171 (6)
N4—H41···O1^i^	0.88 (1)	2.20 (2)	3.008 (6)	153 (4)
N4—H42···O4^ii^	0.88 (1)	2.43 (2)	3.226 (6)	152 (4)
N8—H81···O2^iii^	0.88 (1)	2.27 (1)	3.144 (6)	171 (4)
N8—H82···O4^iv^	0.88 (1)	2.28 (2)	3.127 (7)	161 (6)

Symmetry codes: (i) −*x*+1, *y*+1/2, −*z*+3/2; (ii) *x*−1, *y*, *z*; (iii) *x*+1, *y*, *z*; (iv) −*x*+2, *y*−1/2, −*z*+3/2.
